# Effects of a Virtual Reality-Based Aggression Control Program on Children with Autism Spectrum Disorder: A Case Study

**DOI:** 10.3390/children12020173

**Published:** 2025-01-29

**Authors:** Miran Jung, Jaewon Park

**Affiliations:** 1 Department of Nursing, Baekseok University, Cheonan 31065, Republic of Korea; rcuty@bu.ac.kr; 2 Department of Nursing, Hannam University, Daejeon 34430, Republic of Korea

**Keywords:** aggression, autism spectrum disorder, virtual reality, adaptation, children

## Abstract

**Background/Objectives**: Aggression is a major challenge for children with autism spectrum disorder (ASD), their family members, friends, and teachers because it can pose a threat or harm not only to the children with ASD but also to others. This study is a case study aimed at verifying the effectiveness of a virtual reality-based aggression control program for children with ASD. **Methods**: The participants were two children (one was a 10-year-old boy and the other was a 6-year-old girl) who participated in the ACAA (Aggression Replacement Training for Children and Adolescents with ASD) Program for eight sessions over three weeks. **Results**: The frequency (C1: 48 → 3; C2: 32 → 3) and severity of aggressive behaviors in both participants decreased after the intervention compared to before. Additionally, overall problematic behaviors were also reduced after the intervention (C1: 85 → 70; C2: 87 → 64). Furthermore, both participants demonstrated a slight increase in their levels of adaptation (C1: 17 → 20; C2: 16 → 18). **Conclusions**: The effectiveness of the ACAA program has been demonstrated in reducing levels of aggression in children with ASD. Therefore, the ACAA program may contribute to helping aggressive children with ASD live in harmony with others in society and promote independence.

## 1. Introduction

Autism spectrum disorder (ASD) is a neurodevelopmental disorder. It is characterized by persistent deficits in social interactions and communication and persistently unusual behavior, activities, or interests [1]. Due to the heterogeneity of ASD symptoms and severity, the age at which ASD is diagnosed varies [2]. However, it is estimated that approximately 0.6% of the world’s population suffers from ASD [3]. The number of children with ASD has increased, according to a report from the Autism and Developmental Disabilities Monitoring Network [4].

ASD has several medical, psychological, and behavioral co-occurring symptoms such as feeding or sleep problems, seizures, anxiety, depression, or aggression [2]. Among them, aggression is a major challenge for individuals with ASD and their caregivers because it prevents individuals with ASD from functioning independently and interacting harmoniously with others within society [2,5]. Aggression includes verbal aggression (e.g., cursing, yelling), physical aggression (e.g., biting, hitting, scratching, throwing objects), and self-injurious behaviors (e.g., head banging, hitting or biting self) [6,7]. It is estimated that 17% to 68% of individuals with ASD show aggression [8,9]. Aggression is more common in children than adults among individuals with ASD [8]. Age was shown to be a significant predictor of aggression in children with ASD [5,10,11]. In children, the younger they were, the more aggressive they were [5,11]. One study estimated rates of aggression to be 55% for children aged 6 to 8, 50% for children aged 9 to 11, 40% for children aged 12 to 14, and 25% for children aged 15 and older [5].

Meanwhile, it is suggested that anger and anxiety could provoke aggression in children with ASD [2,9]. Brown et al. [10] reported that anger dysregulation was significantly correlated with aggression. ASD children with anxiety had 4.2 times higher levels of aggression toward others compared to children without anxiety [12]. Specialized teachers reported that children with ASD expressed aggression as an unconscious response to reduce anxiety [13]. Overload or change of sensory stimuli was also a trigger of aggression in children with ASD [9,13]. ASD children who showed greater sensitivity to sensory stimuli were more likely to develop aggression [11]. Aggressive children with ASD can threaten or cause harm not only to themselves but also to their family, friends, and teachers [6,14]. Due to aggressive behaviors, parents felt negative emotions such as frustration during the parenting process [15]. Their peers and teachers experienced tension and distress at school [16]. As a result, aggressive children with ASD may be limited in terms of participating in social activities, attending class, and playing with friends [5,7,14]. This may cause them to lose opportunities to learn social adaptive skills and to fit into society [5,7,13,14]. Specifically, aggressive children with ASD were found to have significantly lower levels of adaptive skills for socialization than those without aggression [11,14].

Several non-pharmacological interventions (e.g., functional behavior assessment, reinforcement strategies, and functional communication training) in combination with pharmacological treatment are used to regulate aggression in children with ASD [6]. These non-pharmacological interventions focus on behavioral aspects to increase desirable behaviors and decrease harmful behaviors [6]. On the other hand, aggression replacement training (ART) is a multi-dimensional intervention to control aggression based on cognitive behavioral theory [17,18]. ART explains that aggression is a complex phenomenon that includes behavioral, emotional, and cognitive components [17]. ART also explains that aggressive children and adolescents are weak in intrapersonal, interpersonal, and social-cognitive skills for prosocial behavior. They also lack the skills to effectively respond to anger or someone’s instructions. As a result, they easily become aggressive rather than responding appropriately to demands or conflict [17]. So, ART includes three components based on the characteristics of aggressive children and adolescents: the behavioral component of social skills training to educate prosocial behavior; the affective component of anger control training to suppress anger by identifying cues and using anger reducers such as deep breathing or imagining a peaceful scene; and the cognitive component of moral reasoning for training to consider justice and the rights of others [17].

In several previous studies, the effects of ART have been reported. Specifically, Hardoni et al. [19] reported a significant decrease in aggression among adolescents after ART intervention. Currie and colleagues [20] administered an ART intervention to 20 youth offenders involved in violence. A significant reduction in aggression level was observed following the intervention [20]. Kaya and Buzlu [18] administered ART to 65 juvenile offenders. After ART, the ART group showed significant reductions in their levels of anger and physical aggression, and significant increases in their levels of social skills for problem-solving. A systematic review reported that ART was significantly effective in reducing aggression in children with ASD. Additionally, approximately 50% of the literature included in the analysis found that ART improved participants’ social adaptive skills [21].

In recent years, virtual reality (VR) technology has been actively used in interventions for children with ASD due to its advantages such as safety, repeatability, excitement through visual and auditory stimuli, no spatial and temporal constraints, and cost-effectiveness [13,22,23,24]. Specifically, children with ASD have trouble regulating their behaviors and fitting themselves into the real world [22]. They also feel frustration and anxiety when they experience failure or refusal in interpersonal relationships in the real world [24]. Real-life situations that children with ASD may face can be elaborately and purposively manipulated in VR environments [22,23]. Without feeling frustrated by failure or mistakes, children with ASD can learn target skills needed for everyday life in a real-world context [22]. Additionally, VR is presented as a valuable intervention tool in that it allows children with ASD to strengthen their skills through repetitive practice [22]. VR-based interventions are specifically used to approach social and emotional challenges in children with ASD [23]. A study reported the effectiveness of a VR-based intervention on situation and emotion recognition [25]. Ip et al. [26] applied a VR-based intervention to school-aged children with ASD to verify its effects on emotional skills and social adaptive skills. Results showed that emotional regulation, emotional expression, and social interaction were significantly increased after VR-based intervention.

VR technology has been used in interventions based on a combined approach of cognition and behavior. Pot-Kolder et al. [27] applied cognitive behavioral therapy (CBT)-based VR intervention to participants with paranoid ideation. Cases included in the intervention were structured to include clues that could lead to paranoid thoughts, fears, and behaviors. This was because paranoid thinking was caused by an emotional element such as fear. Alsem et al. [28] developed a VR intervention based on CBT to control aggression in children. The program was designed to help children practice information processing and emotional regulation. In social relationships, emotions play an important role in effective interactions with others [29]. Lorenzo et al. [29] created social situations containing emotional elements such as anger and happiness that children with ASD may encounter in real life. Social situations were presented to children through VR technology. After intervention, the children’s adaptive behavior significantly increased, and maladaptive behavior significantly decreased. The advantage of VR is that it can create realistic complex situations combining cognition, emotion, and behavior [22]. Children can acquire skills by immersing themselves in safe and realistic situations through VR programs.

Despite the effects of ART on aggression control [18,19,20,21], few studies have been conducted on ART intervention among children with ASD. VR-based interventions have been reported to be effective for social adaptive skills [22] and emotional awareness [22,25] in children with ASD, but no interventions have been attempted to control aggression. Accordingly, we set the research questions as follows. The primary research question was the following: is VR-based ART intervention effective in reducing aggression in children with ASD? The secondary research question was the following: is VR-based ART intervention effective in improving adaptation in children with ASD? We developed a VR-based aggression control program for children with ASD to reduce aggression with a theoretical background in ART [17]. The purpose of this study was to conduct a VR-based aggression control program first on children with ASD who showed aggression and confirm its effectiveness. This was based on previous research showing that children aged 6 to 11 were more aggressive than other age groups [5].

## 2. Materials and Methods

### 2.1. Design

This is a case study aimed at verifying the effectiveness of a virtual reality-based aggression control program for children with ASD. The study was designed with consideration of the CARE guidelines provided by the Equator Network [30] from the outset and reported in accordance with these guidelines.

### 2.2. Participants

The participants of this study were school-aged children in Korea who were diagnosed with ASD and exhibited aggressive behaviors. The specific inclusion criteria were as follows: diagnosed with ASD and currently registered as a person with severe disabilities; displaying aggressive behaviors at least once a week within the past six months; having a history of hospital visits or counseling due to aggressive behaviors; without concurrent visual or auditory impairments; willing to participate in the study with parental consent. Even if an individual met the inclusion criteria for this study, they were excluded if they fell under any of the following exclusion criteria. The exclusion criteria were individuals with moderate to severe medical or neurological conditions, those with coexisting physical disabilities such as brain lesions or polio, individuals exhibiting psychotic symptoms, those with a history of moderate to severe head trauma, and those unable to participate in the assessments conducted in this study. Initially, three participants were recruited, but one child and their mother withdrew from the study early due to contracting COVID-19, resulting in a final sample size of two participants. The profiles of the study participants are presented below, and pseudonyms have been used to protect their identities.

#### 2.2.1. Case 1 (Lucky; Male, 10 Years Old) Profile

Lucky is currently a fourth-grade student enrolled in a special education elementary program in Gyeonggi Province, South Korea. He is the eldest of two sons, with a younger sibling three years his junior. Lucky lives with his parents and sibling, and the family home is located within a five-minute walking distance from his school, allowing him to commute on foot each day. Both parents are employed, hold postgraduate degrees, and reported an annual income exceeding KRW 200 million. During weekdays, Lucky’s primary caregivers are his maternal grandparents, who take responsibility for his care and transportation to school and therapy centers. On weekends, his parents assume full responsibility for his education, therapy, and general caregiving.

At the initial interview, Lucky’s K-CARS score was confirmed to be 44, indicating a severe level of autism (Table 1). Lucky was diagnosed with ASD at the age of 2 years and 4 months and first received treatment for developmental delay at 1 year and 6 months. Currently, Lucky is registered with a diagnosis of severe autism. Lucky could respond to questions with basic information such as his name and simple greetings, demonstrating the ability to repeat spoken words, though approximately 50% of his articulation was assessed as unclear. Most of his speech consisted of incomprehensible vocalizations, with receptive language skills observed to be stronger than expressive language abilities. Additionally, Lucky showed partial proficiency in reading and writing Korean.

He currently exhibits aggressive behaviors, such as screaming, scratching, biting, pounding on doors, and throwing objects, which occur more than once a week and affect both himself and others. Due to these problematic behaviors, he has been undergoing continuous outpatient pharmacological treatment and has received behavioral therapy twice (at ages 5 and 6), along with more than seven counseling sessions. He is currently on risperidone 4 mg once daily (OD), which he began taking at age 4 for sleep disturbances and has since been increased to the current dosage.

The behavior deemed most severe by the respondent was scratching and biting both himself and others. It was reported that his aggressive behavior escalated after an incident during social training at an external treatment facility when he was 5, where he was bitten and hit by another child with developmental disabilities. The response regarding Lucky was provided by his mother, who, upon observation, was noted to have numerous scars on her face, hands, and arms, as well as 12 bandages covering injuries. She stated that these injuries were caused by Lucky. Furthermore, the maternal grandmother sustained a fracture in her left wrist while attempting to prevent Lucky’s aggressive behavior, and the maternal grandfather also had scars on his face and hands. All caregivers viewed Lucky’s aggressive behavior as severe, and this issue was considered a critical concern at both his school and treatment center. Lucky’s caregiving is shared among four individuals: two maternal grandparents during weekdays, his mother in the mornings and evenings, and both parents on weekends. The caregiving load appears to be well-distributed, and the family is considered to have a strong support system.

#### 2.2.2. Case 2 (Vicky; Female, 6 Years Old) Profile

Vicky is a female child currently enrolled in the first grade of a special education elementary school located in Incheon, South Korea. She is the eldest of two children, having a younger brother who is one year younger. Vicky lives with her parents and brother. The distance between her home and the special education school is relatively short, approximately a 15-min drive, and she commutes to school daily by school bus. Her father works as a salaried employee, serving as the primary breadwinner, while her mother takes full responsibility for the care of the children, including Vicky’s education and therapy. Both parents hold at least a university degree, and the family reported an average annual income of approximately KRW 50 million.

At the initial interview, her score on the K-CARS was confirmed to be 42 (Table 1), indicating a severe level of autism. Lucky was diagnosed with ASD at the age of 4 years and 8 months, and she first received treatment for developmental delay at the age of 3 years and 11 months. She is currently registered with a diagnosis of severe autism. Vicky was able to answer simple questions, such as stating her name, favorite things, and greetings. She could engage in echolalia and her articulation was relatively good, with only about 10% of her speech judged to be unclear. Overall, she rarely initiated verbal communication and exhibited minimal solitary muttering. It was observed that her receptive language abilities were stronger than her expressive language skills. She had not yet demonstrated the ability to read or write in Korean.

She currently exhibits aggressive behaviors such as yelling, hitting, banging her head on the floor, striking objects, and throwing items more than once a week, which are primarily directed toward herself or her surroundings rather than others. When aggression is directed toward others, it predominantly targets her younger sibling. She has been receiving treatment for aggression since the age of five, beginning with risperidone therapy. At present, she is taking risperidone 3 mg and aripiprazole (Abilify) 2 mg OD. She has no history of behavioral therapy but has undergone more than five sessions of counseling for aggression.

The most severe behavior among the child’s problematic actions was reported to be self-injury, with increasing frequency of behaviors such as hitting her head or banging it against the floor. However, no specific event could be recalled as a trigger. The respondent to questions regarding Vicky was her mother. Upon observation, no physical trauma was noted in the mother, but she expressed significant feelings of depression and mentioned that she was undergoing psychiatric treatment for it. Although there was no visible damage in the home, the mother reported living in a high-rise apartment and frequently receiving complaints from the downstairs neighbors due to noise. The mother considered Vicky’s aggressive behavior to be severe, while the father assessed the issue as moderate. Both the school and the treatment center identified self-injury as one of the primary concerns. Vicky’s primary caregiver was her mother, who was responsible for the majority of her children’s education, treatment, and upbringing. As a result, the burden and fatigue she experienced was considerable. Although the family’s support system was not extensive, occasional help was provided by grandparents and an aunt.

### 2.3. Measures

#### 2.3.1. Virtual Reality Based ACAA (Aggression Replacement Training for Children and Adolescents with Autism Spectrum Disorder) Program

This program was developed based on ART with the aim of reducing aggression in children with ASD by decreasing their sensitivity to sensory information, enhancing the acquisition of basic social skills, and alleviating anxiety and anger. To implement the intervention through virtual reality, the program was designed to be integrated within a head-mounted display, with Oculus Quest 2 selected as the device of choice due to its wireless capability and stable fit, ensuring ease of use and comfort during wear.

**Figure 1:**
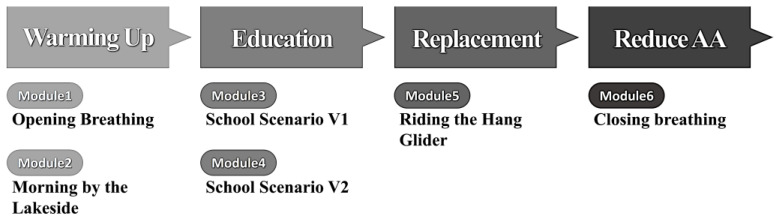
Structure of the ACAA program.

Based on a comprehensive review of the relevant literature, including systematic reviews and focus group interviews, a structured process for the program was developed, consisting of multiple stages: warm-up, education, replacement, and anger and anxiety (AA) reduction [13,17,31,32]. Initially, three content modules were conceived for each stage. However, during program development advisory meetings, it was concluded that extending the duration of each session could hinder engagement, particularly given the short attention span of the target participants. As a result, the final program was streamlined to focus on six essential content modules that are most crucial for reducing aggression (Figure 1). Additionally, considering the developmental differences between children and adolescents, the first version of the ACAA program was designed to focus on children as the primary target group.

In the Warm-up phase, two components were selected based on various studies demonstrating their effectiveness in reducing aggression: diaphragmatic breathing [33,34,35] and exposure to natural environments [36,37]. The natural environment component was designed as a scene in which participants view scenic landscapes while riding on a boat across a lake. In the Education phase, the influence of social adaptation on ASD children’s aggression was considered [38], and two simple educational modules were developed to teach interpersonal skills. These modules were based on two episodes designed to increase tension and stress, focusing on relationships with friends and teachers, respectively. For the Replacement phase, the content was built around an activity that embodies the transition to a new experience, which can help decrease aggression [17,39]. This involved the exhilarating experience of hang gliding through the sky. Lastly, the AA Reduction phase revisited the breathing exercises from the initial phase, concluding the activity with this calming technique. Each module was designed to last 2 to 5 min, ensuring that the entire four-phase program did not exceed 15 min.

**Figure 2:**
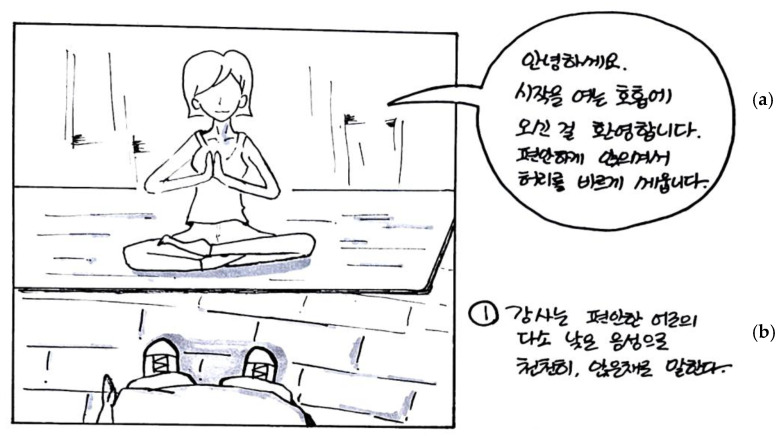
Example of the ACAA content storyboard. The translation is as follows: (**a**) Hello. Welcome to Opening Breathing. Please sit down comfortably and keep your back straight.; (**b**) The instructor sits down and speaks slowly and in a relaxed tone of voice.

The principal investigator directly developed the script and storyboard for this study. The drafted script was refined and finalized with the consultation of one research assistant experienced in simulation program development, a professor, and an expert in autism research. The storyboard was hand-drawn by the principal investigator and further enhanced with the assistance of a research assistant to ensure a more natural and realistic representation (Figure 2). The initially completed script and storyboard were revised and supplemented through meetings with the video production team, leading to the final version. The VR content was produced using live action, and the entire process, from casting actors, securing locations, props, and equipment, to the actual filming, took a total of two months. Following the initial filming, additional shooting was conducted based on subsequent meetings, with the total time for filming and video editing extending to six months.

#### 2.3.2. Behavior Problems Inventory

To evaluate the problem behaviors of children with ASD who were participants in this study, the Korean version of the Behavior Problems Inventory (BPI) was utilized. This tool is a behavior assessment instrument based on informant reports. In this study, it was administered as a caregiver-reported measure, consisting of a total of 52 items, including 15 items for assessing self-injurious behaviors, 25 items for stereotypic behaviors, and 12 items for aggressive/destructive behaviors [40].

#### 2.3.3. The Child Behavior Checklist for Ages 6–18 (Korean Version); K-CBCL 6-18

In this study, the Korean version of the Child Behavior Checklist for Ages 6–18 (K-CBCL 6-18) was utilized to assess children’s behavior and adaptation levels. This tool, originally developed by Achenbach and Rescorla [41] and later standardized by Oh et al. [42], is classified into two main scales: the problem behavior syndrome scale and the adaptive functioning scale. The problem behavior syndrome scale is considered clinically significant when the T-score is 64 or higher, indicating notable behavioral issues. Conversely, the adaptive functioning scale is deemed clinically significant for adaptation difficulties when the T-score is 36 or lower. To use this tool for the study, it was purchased through the ASEBA institution, which holds the licensing rights for its use.

### 2.4. Procedure

Consent was obtained from participants and their parents for participation in the study. Prior to initiating the main intervention, a two-week adaptation period was provided for the participants to familiarize themselves with the VR device, specifically the head-mounted display. The total application of the ACAA program consisted of eight sessions conducted three times per week over a span of three weeks.

Participants sequentially progressed through the four stages of the ACAA program. As the content for the warm-up and education stages included two activities per session, each session involved two cycles, and alternative content was used for the initial cycle. The program was conducted in a stepwise manner according to the stages. The duration of each session was adjusted based on the child’s condition, ensuring that it did not exceed one hour.

With parental consent, portions of the program were video recorded. Additionally, the assessment of the two participants’ behaviors, including evaluations of problem behaviors and child and adolescent behavioral assessments, were conducted by the same researcher at pre-, mid-, and post-intervention stages. Data collection for the participants was conducted under the approval of the IRB until February 2022.

### 2.5. Ethical Considerations

Before starting the study, we obtained Institutional Review Board approval (IRB No. BUIRB-202008-HR-016). The research was guided by the Declaration of Helsinki, including respect for autonomy, justice, non-maleficence, and beneficence [43]. Written informed consent was obtained solely from parents who were fully informed about the importance of anonymity and voluntary participation for both data collection and program intervention. Additionally, participants and their parents were informed that they could withdraw from participation in the study at any time without penalty, and that aggressive behaviors would be measured via video recordings at the beginning, middle, and end of the study. In appreciation for their contributions, participants were compensated with gift vouchers, taking into account the time committed to the research.

## 3. Results

### 3.1. Changes in Aggressive Behaviors

**Figure 3:**
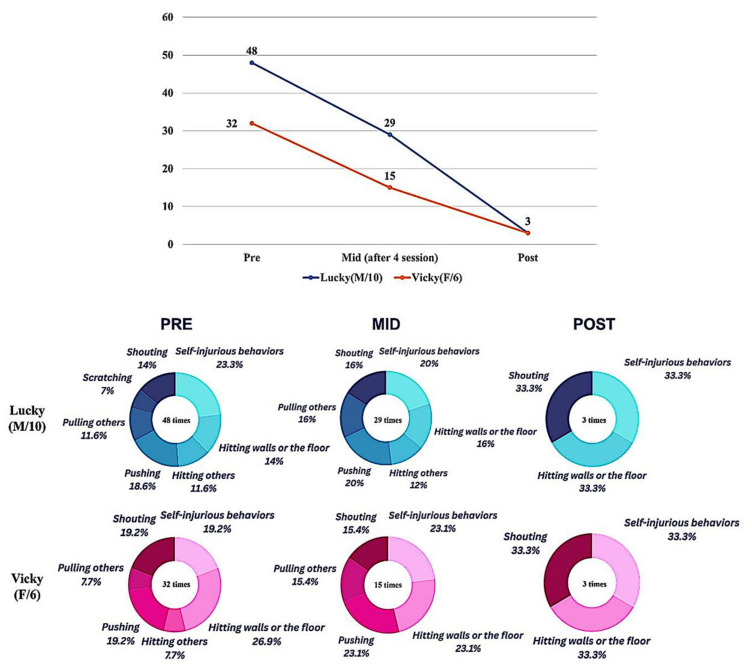
Changes in the frequency of aggressive behaviors at pre-, mid-, and post-intervention.

The frequency of aggressive behaviors in the two study participants was assessed before, during, and after the ACAA program intervention. As shown in Figure 3, both participants demonstrated a significant reduction in the frequency of aggressive behaviors following the intervention compared to before. Specific behaviors such as self-injurious behaviors, hitting walls or floor, shouting, and physical aggression toward others (pushing, pulling or hitting others, scratching, and so on) showed marked decreases (Figure 3).

Furthermore, an evaluation of the participants’ aggressive behaviors before and after the program intervention, using the BPI problem behavior assessment tool, revealed a significant reduction in both the frequency and severity scores in the two domains of self-injurious behavior and aggressive/destructive behavior for both participants after the intervention compared to before the intervention. Additionally, the K-CBCL 6-18 tool was used to assess the participants’ aggressive behaviors at pre-, mid-, and post-intervention stages of the ACAA program. The results also indicated that aggressive behaviors decreased in both participants after the implementation of the ACAA program compared to before its implementation (Table 2).

### 3.2. Changes in Other Problem Behaviors

To examine changes in problem behaviors other than aggressive behaviors, the results obtained using the BPI tool revealed that both participants showed a decrease in the severity and frequency of stereotypic behaviors after the intervention compared to before the intervention (Table 3). Additionally, overall problem behaviors were measured using the K-CBCL 6-18 tool. The results showed that the total problem scores for both participants remained within the clinical range pre-, mid-, and post-intervention. However, both participants showed a decrease in total problem scores after the intervention compared to before. Based on T-scores, Case 1 showed a reduction of 15 points, and Case 2 showed a reduction of 23 points. Also, the internalizing total scores, including anxious/depressed, withdrawn/depressed, and somatic complaints, as well as the externalizing total scores, including rule-breaking behavior and attention problems, decreased for both participants after the intervention compared to before. Among these, the reduction in externalizing total scores was more significant, with Case 1 showing a decrease of 11 points and Case 2 showing a decrease of 36 points based on T-scores (Table 3).

### 3.3. Changes in Adaptation Level

Using the K-CBCL 6-18 tool, a comparison of the total adaptation scale scores before and after the program intervention revealed a slight increase for both participants (Case 1: 17 → 20 points, T-scores; Case 2: 16 → 18 points, T-scores). However, prior to the intervention, the percentage scores for the total adaptation scale of both participants were below 1%. Similarly, even after the intervention, the total adaptation scale scores for both participants remained below 1%, indicating that their adaptation levels were within the lowest 1% of the standardized reference population (Table 4). Therefore, when considering the entire standardized reference population, the effect of this intervention on the adaptation levels of the two participants was found to be minimal.

## 4. Discussion

This study was conducted to investigate the effectiveness of ART- and VR-based intervention on aggression in children with ASD. The ACAA program, which was developed in this study, reduced the level of aggression in children with ASD. The level of adaptation increased slightly, but the significance was marginal. Although the current study was a two-participant case study, this study is significant in that it was the first ART- and VR-based intervention to reduce aggression in children with ASD. Several studies reported ASD children at a young age have higher levels of anger dysregulation, physical aggression, and aggressive behavior toward others [5,10]. Therefore, this study, which confirmed the effectiveness of the ACAA program for relatively young children aged 6 to 11, is meaningful.

The ACAA program was developed based on ART [17]. It included anger control training to alleviate anger and anxiety and social skills training to educate interpersonal skills. In the warm-up phase, participants took deep breaths and experienced the peaceful natural environment of boating on a calm lake. It made participants calm and alleviated their anger and anxiety. Children with ASD experience tension and stress when peers talk to them or when they are exposed to a noisy classroom environment. During the education phase, participants were exposed to situations that could be experienced at school. Through this stage, children learned interpersonal skills and were trained to reduce their sensitivity to sensory stimulation. The levels of aggressive behaviors in both participants decreased after the ACAA program compared to before. This is consistent with previous findings showing significant reductions in levels of aggression following ART intervention in adolescents with violence [19,20].

Hardoni et al. [19] suggested that the reduction in participants’ aggression after ART intervention was due to structured training in the three components of ART. Previous research has shown significant reductions in adolescents’ anger levels as well as aggression levels following ART intervention [18,19]. Kaya and Buzlu [18] found that after ART intervention, not only did adolescents’ levels of anger decrease, but their levels of anger control also increased. It was interpreted that participants used what they learned in anger control training and social skills training when faced with stressful situations [18]. Based on these results, the mechanisms of ART are not only effective in reducing aggression in children and adolescents but also contribute to reducing aggression in children with ASD. This suggests that encompassing behavioral, emotional, and cognitive factors is important in reducing aggression. Nevertheless, caution is needed in interpreting the results, because this study had a limited number of participants and was not an experimental design that included a control group. Future research with experimental designs including large samples and control groups is needed to determine the effectiveness of the ACAA program.

VR technology creates a realistic environment for subjects through visual and auditory stimuli [24,25]. Real-life scenes in VR situations can help participants feel familiar with the learning contents [22]. The ACAA program provided components for anger and anxiety training and social skills training in a realistic context. Participants could learn by immersing themselves in realistic situations. The module of riding a hang glider was added in the replacement stage. It helped participants replace aggression with new experiences. Participants were immersed in the intervention using VR technology to simulate boating on a lake, receiving guidance from a teacher, and hang-gliding experiences. Additionally, for children with ASD, individualized approaches are more appropriate than group approaches [9] due to their interaction difficulties and high sensitivity. Because the ACAA program was a home-based intervention, it allowed an individualized approach to participants [23]. It is expected that participants participated in the ACAA program with a sense of safety, free from social tension or emotional suffering [22,23]. Education delivered in 2D requires students to imagine educational situations. On the other hand, learning through VR improves real-life applications because it allows learners to feel and simulate being in the real world [22]. This may have contributed to reducing aggression in participants.

Nevertheless, specialized teachers were concerned that children with ASD might be reluctant to wear devices for VR programs [13]. In fact, in our study, it took participants two weeks to become familiar with the device for the VR program. The reason was that participants refused to wear a head-mounted display device. Therefore, research is needed to develop more convenient devices for VR programs and strategies to reduce aversion to devices. There is also a need to expand beyond VR technology to the interventions using multi-sensory extended reality.

In this study, the ACAA program was more effective in reducing physical aggression toward others (e.g., pulling, pushing, or hitting others). ART was also more effective in reducing physical aggression in previous studies [18,20]. Because aggression appears as an externalized problem behavior, it has a negative impact on family functioning in that it can cause harm or pose a threat to others [15]. High levels of aggression interfere with the social activities of family and siblings and cause negative feelings about parenting and conflict between spouses [15]. This is interpreted to be because parents of ASD children who are highly aggressive spend a lot of energy and time monitoring and controlling their children’s acting-out behaviors [15]. Additionally, it can be inferred that the children’s aggressive behaviors toward others can cause social withdrawal in children with ASD and family members. Therefore, the ACAA program is expected to contribute to improving family functioning by reducing physical aggression toward others in children with ASD.

The result showed both participants’ adaptation scores slightly increased after the ACAA program compared to before. However, the pre-and post-intervention percentage scores on the total adaptation scale for both participants were within the lowest 1% of the standardized reference population. Therefore, the increase in participants’ adaptation score in this study may ultimately be interpreted as meaningless. The reason why the level of adaptation did not increase significantly is interpreted to be because moral reasoning of ART was not included in the ACAA program. In moral reasoning of ART, children are introduced to the situation to induce cognitive conflict [17]. Children control cognitive distortions and develop moral maturity through discussions about justice and the rights of others [17]. Children learned what not to do and what to do through anger management training and social skills training [17]. Children show behavioral changes through cognitive changes [44]. In the previous studies applying an ART intervention to youth with violence problems, ART interventions were significantly effective in reducing aggression and enhancing social adaptive skill [18,20,44]. While these previous studies included all three components of ART [18,20,44], the ACAA program lacked moral reasoning. As a result, it was determined that changes were induced at the level of aggression, but no significant changes were induced at the level of adaptation. Therefore, future research will need to develop the ACAA program to include moral reasoning and verify its impact on the level of adaptation. VR allows children with ASD to learn social adaptive skills more quickly in safer environments [25]. We propose developing a program that involves educators in a VR environment and interaction between educators and children with ASD. This design would help strengthen the effects of the ACAA program by adding moral reasoning.

Moreover, while participants in previous studies were violent youth, participants in this study were children with ASD. Children with ASD are characterized by unusual communication, interaction, behavior, and interests [1]. The participants in this study were children with severe levels of ASD. This can also be confirmed in the pre-intervention adaptation scores of the two participants. This may have influenced the results because the adaptation levels of the two participants were different from those of children without ASD. Therefore, in future studies, it is necessary to confirm the effectiveness of the ACAA program by applying it to children with mild or moderate levels of ASD.

The goals of interventions for children with ASD are to alleviate symptoms, minimize problematic behaviors, and enhance independence by improving adaptive social skills [9]. Moreover, aggression cannot be controlled with one-time intervention; it must be reduced through ongoing intervention [13]. The ACAA program is the first aggression control program based on ART and VR technologies. The effectiveness of the ACAA program was demonstrated in reducing levels of aggression in children with ASD. Therefore, the results of this study are expected to provide a foundation for research on controlling aggression in children with ASD. Further, the ACAA program may contribute to helping aggressive children with ASD live in harmony with others in society and promote independence.

This study has several limitations. This study was designed as a case study without a control group, and only two children with ASD participated in the research. Results of this study showed the positive impact of the ACAA program on aggression and adaptation in children with ASD. Nevertheless, it is difficult to generalize the research findings due to the absence of comparative criteria and the small sample size. In order to generalize the effects of the ACAA program, follow-up research to complement the limitations of this study must be conducted. Additionally, since the results of this study suggest only short-term effects, additional research on long-term effects is needed to generalize the results. Another limitation is that the ACAA program lacked contents for moral reasoning training, the third component of ART for cognitive training. It is needed to develop programs that strengthen moral reasoning elements according to the level of intelligence of the children with ASD. Lastly, the outcome variables in this study were measured through video recording. Because the video recordings did not last 24 h, participants’ aggressive behaviors were not assessed over a 24 h period. Although outcome variables were measured by the same researcher to ensure consistency of results, more sophisticated methods are needed to measure outcome variables more accurately in future studies.

Despite the limitations of this study, our findings may contribute to research and clinical practice to alleviate aggression and increase social adaptation in children with ASD. Recommendations for future studies are as follows. First, experimental studies with large samples and control groups are required to verify the effect of the ACAA program in children with ASD. Second, research should be conducted to test the long-term effects of the ACAA program. Third, studies are necessary to verify the ACAA program’s effects by applying it not only to children with severe symptoms of ASD, but also to children with mild and moderate symptoms of ASD. Lastly, research to verify the effects of the ACAA program by applying it to adolescents with ASD would be helpful.

## Figures and Tables

**Table 1:** K-CARS results of two cases.

CASE 1 (Lucky)							
Interpersonal Relationship	Imitation	Emotional Response	Use of Body	Use of Objects	Adaptation to Change	Visual Response	Auditory Response
3	3	3	3	3	3	3	3
Sensory Response	Fear and Anxiety	Verbal Communication	Non-verbal Communication	Activity Level	Intelligence and Cognitive Ability	General Impressions	Total Score
3	3	3	3	3	2	3	44.0
CASE 2 (Vicky)							
Interpersonal Relationship	Imitation	Emotional Response	Use of Body	Use of Objects	Adaptation to Change	Visual Response	Auditory Response
3	2	3	3	3	3	3	2
Sensory Response	Fear and Anxiety	Verbal Communication	Non-verbal Communication	Activity Level	Intelligence and Cognitive Ability	General Impressions	Total Score
3	3	3	2	3	3	3	42.0

**Table 2:** Changes in aggressive behavioral scores measured at pre-, mid-, and post-intervention using the BPI and K-CBCL 6-18 tools.

Measurement Tool	Aggressive Behaviors	Case 1 (Lucky, M/10)			Case 2 (Vicky, F/6)		
		Pre	Mid	Post	Pre	Mid	Post
BPI	Self-injurious behaviors severity	17	-	16	5	-	2
	Self-injurious behaviors frequency	17	-	9	6	-	2
	Aggressive/Destructive behaviors severity	15	-	7	10	-	7
	Aggressive/Destructive behaviors frequency	17	-	7	10	-	7
K-CBCL 6-18	Aggressive behaviors	69 (97) *	67 (95) *	57 (77) *	77 (99) *	74 (99) *	50 (50) *

* T Score (%).

**Table 3:** Changes in other problem behavioral scores measured by the BPI and the K-CBCL 6-18 at pre-, mid-, and post-intervention.

Measurement Tool	Problem Behaviors	Case 1 (Lucky, M/10)			Case 2 (Vicky, F/6)		
		T Score (%)			T Score (%)		
		Pre	Mid	Post	Pre	Mid	Post
BPI	Stereotypic behaviors severity	45 *	-	34 *	31 *	-	25 *
	Stereotypic behaviors frequency	67 *	-	34 *	32 *	-	26 *
K-CBCL 6-18	Total Problem Behavior	85 (99)	80 (99)	70 (98)	87 (99)	83 (99)	64 (92)
	Internalizing	68 (96)	68 (96)	64 (93)	63 (90)	63 (90)	56 (73)
	Externalizing	68 (97)	65 (94)	57 (75)	90 (100)	83 (99)	54 (65)
	Anxious/Depressed	67 (95)	67 (95)	65 (93)	55 (71)	58 (80)	55 (71)
	Withdrawn/Depressed	66 (95)	66 (95)	66 (95)	73 (99)	73 (99)	63(90)
	Somatic Complaints	66 (94)	66 (94)	55 (69)	53 (62)	50 (26)	50 (26)
	Rule-Breaking Behavior	65 (94)	59 (81)	54 (64)	71 (99)	69 (98)	61 (87)
	Aggressive Behaviors	69 (97)	67 (95)	57 (77)	77 (99)	74 (99)	50 (50)
	Social Immaturity	68 (97)	68 (97)	68 (97)	70 (98)	70 (98)	68 (96)
	Thought Problems	74 (100)	72 (100)	70 (99)	75 (100)	74 (100)	70 (99)
	Attention Problems	90 (100)	90 (100)	86 (100)	87 (100)	84 (100)	79 (99)
	Other Problems	77 (100)	75 (100)	71 (99)	69 (97)	70 (98)	62 (89)

* Raw scores measured by the BPI.

**Table 4:** Adaptation level changes measured by the K-CBCL 6-18 at pre- and post-intervention.

	Case 1 (Lucky, M/10)		Case 2 (Vicky, F/6)	
	Pre	Post	Pre	Post
Total Adaptation Scale	17 (1%)	20 (1%)	16 (0%)	18 (1%)

## Data Availability

The data presented in this study are available on request from the corresponding author due to participant privacy and ethical reasons.

## Abbreviations

The following abbreviations are used in this manuscript:

ASD	Autism spectrum disorder
ART	Aggression replacement training
VR	Virtual reality
ACAA	Aggression replacement training for children and adolescents with autism spectrum disorder
